# 5-Bromo-17-nitro-26,28-prop-2-en­oxy-25,27-dipropoxycalix[4]arene

**DOI:** 10.1107/S160053680900659X

**Published:** 2009-02-28

**Authors:** Catharina Hippius, Frank Würthner, Michael Bolte

**Affiliations:** a Institut für Organische Chemie, Universität Würzburg, Am Hubland, 97074 Würzburg, Germany; b Institut für Anorganische Chemie, J. W. Goethe-Universität Frankfurt, Max-von-Laue-Strasse 7, 60438 Frankfurt/Main, Germany

## Abstract

Mol­ecules of the title compound, C_40_H_42_BrNO_6_, are located on a crystallographic twofold rotation axis. As a result, the nitro group and bromine residue are mutually disordered with equal occupancies. The prop­oxy-substituted aromatic rings are close to parallel to each other [dihedral angle = 21.24 (1)°], whereas the propen­oxy-substituted rings enclose a dihedral angle of 70.44 (1)°. The dihedral angles between the methyl­ene C atoms and the aromatic rings shows that the propen­oxy substituted rings are bent away from the calixarene cavity [dihedral angle between the planes = 35.22 (8)°], whereas the prop­oxy-substituted rings are almost perpendicular [79.38 (10)°] to the plane of the methyl­ene C atoms.

## Related literature

For related literature on calix[4]arenes, see: Asfari *et al.* (2001 ▶); Böhmer (1995 ▶); Gutsche (1998 ▶); Mandolini & Ungaro (2000 ▶). For the synthesis of the title compound, see: Sansone *et al.* (2004 ▶).
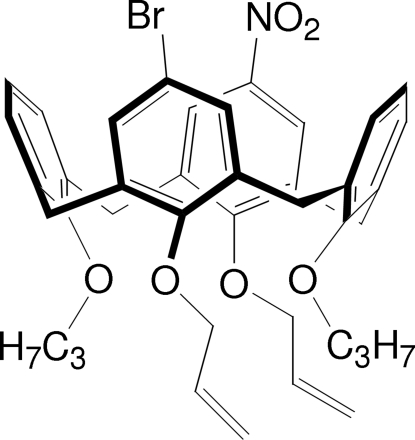


## Experimental

### 

#### Crystal data

C_40_H_42_BrNO_6_
*M* *_r_* = 712.66
Monoclinic, 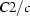

*a* = 25.001 (3) Å
*b* = 8.4963 (14) Å
*c* = 19.909 (3) Å
β = 121.530 (8)°
*V* = 3604.6 (9) Å^3^
*Z* = 4
Mo *K*α radiation
μ = 1.19 mm^−1^
*T* = 173 K
0.26 × 0.12 × 0.11 mm

#### Data collection

Stoe IPDS-II two-circle diffractometer
Absorption correction: multi-scan (*MULABS*; Spek, 2009 ▶; Blessing, 1995 ▶) *T*_min_ = 0.748, *T*_max_ = 0.881
9833 measured reflections
3366 independent reflections
1538 reflections with *I* > 2σ(*I*)
*R*_int_ = 0.081

#### Refinement

*R*[*F* ^2^ > 2σ(*F* ^2^)] = 0.065
*wR*(*F* ^2^) = 0.130
*S* = 0.92
3366 reflections
286 parameters
47 restraints
H-atom parameters constrained
Δρ_max_ = 0.26 e Å^−3^
Δρ_min_ = −0.29 e Å^−3^

### 

Data collection: *X-AREA* (Stoe & Cie, 2001 ▶); cell refinement: *X-AREA*; data reduction: *X-AREA*; program(s) used to solve structure: *SHELXS97* (Sheldrick, 2008 ▶); program(s) used to refine structure: *SHELXL97* (Sheldrick, 2008 ▶); molecular graphics: *XP* in *SHELXTL-Plus* (Sheldrick, 2008 ▶); software used to prepare material for publication: *SHELXL97*.

## Supplementary Material

Crystal structure: contains datablocks I, global. DOI: 10.1107/S160053680900659X/at2726sup1.cif

Structure factors: contains datablocks I. DOI: 10.1107/S160053680900659X/at2726Isup2.hkl

Additional supplementary materials:  crystallographic information; 3D view; checkCIF report

## Figures and Tables

**Fig. 1.**
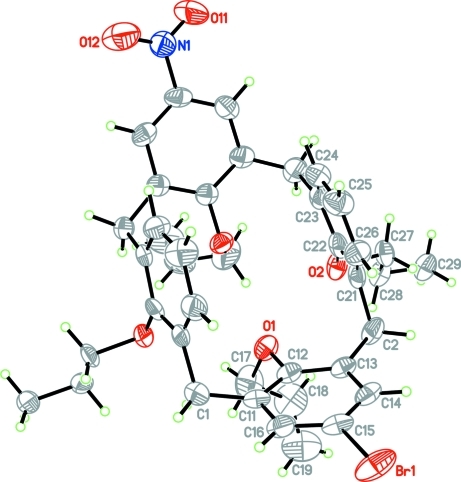
Perspective view of the title compound with the atom numbering; displacement ellipsoids are at the 50% probability level. Only the major occupied site of the disordered moieties is shown. [Symmetry operator for generating equivalent atoms: 1 - x, y, 1/2 - z. ]

**Fig. 2.**
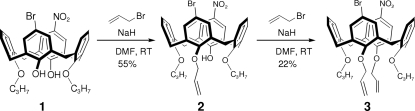
The formation of the title compound.

## supplementary crystallographic information

### Comment

Calix[4]arenes are macrocycles which provide an ideal scaffold to preorganize functional units for application in catalysis or molecular recognition. They are available in large quantities and can be easily modified by selective reactions involving the wide or narrow rim of the molecule (Asfari *et al.*, 2001; Mandolini & Ungaro, 2000; Gutsche, 1998; Böhmer, 1995). Accordingly, compound 1 was obtained by bromination of 5-mononitro-26,28-dipropyloxycalix[4]arene in CH_2_Cl_2_ with 59% yield (Sansone *et al.*, 2004). Subsequent reaction of 1 with allyl bromide and NaH in DMF at room temperature afforded the respective monoalkylated derivative 2. Further reaction of compound 2 with a large excess of allyl bromide afforded compound 3 with 22% yield.

Molecules of the title compound 3 (Fig. 1) are located on a crystallographic twofold rotation axis. As a result of that, the nitro group and bromine residue are mutually disordered. The propoxy substituted aromatic rings are almost parallel to each other [dihedral angle 21.24 (1)°], whereas the propenoxy substituted rings enclose a dihedral angle of 70.44 (1)°. The dihedral angles between the methylene C atoms and the aromatic rings shows that the propenoxy substituted rings are bent away from the calixarene cavity [dihedral angle between the planes 35.22 (8)°] whereas the propoxy substituted rings are almost perpendicular [79.38 (10)°] to the plane of the methylene C atoms.

### Experimental

5-Mononitro-26,28-dipropyloxycalix[4]arene was synthesized according to literature (Sansone *et al.*, 2004).  Under an argon atmosphere 29 mg (0.045 mmol, 1 equiv.) of compound 2 and 26 mg (1.12 mmol, 25 equiv.) of NaH were suspended in 2 ml of dry DMF and stirred for some minutes. Afterwards, 135 mg (1.12 mmol, 25 equiv.) of allyl bromide in 0.7 ml DMF were slowly added to the mixture and the latter stirred for additional 12 h at room temperature. Subsequently, the reaction suspension was slowly stirred into a mixture of 20 ml CH_2_Cl_2_ and 10 ml 1 N HCl. The obtained organic phase was separated, washed with water and brine and dried over MgSO_4_. The solvent was evaporated and the resulting crude product was purified by column chromatography with CH_2_Cl_2_/pentane 40:60 and subsequent crystallization from CHCl_3_/methanol. Compound 3 was obtained as white crystals (7 mg, yield 22%). C_40_H_42_BrNO_6_ (712.67). Mp = 203–206°C. CH_2_Cl_2_/Hexan 40:60; *R*_f_=0.30. ^1^H-NMR (400 MHz, CDCl_3_, TMS, 25°C) δ (p.p.m.): 7.80 (s, 2H; Ar-*H*); 6.96 (s, 2H; Ar-*H*); 6.50–6.39 (m, 6H; Ar-*H*); 6.37 - 6.26 (m, 2H; AllylC=*H*); 5.21–5.16 (m, 4H; AllylC=*H*_2_); 4.66 and 4.64 (dt, 2H, ^3^J=6.0 Hz, ^4^J=0.98 Hz; *O*—*CH*_2_Allyl); 4.52 and 4.50 (dt, 2H, ^3^J=6.5 Hz, ^4^J=1.1 Hz; *O*—*CH*_2_Allyl); 4.65 and 3.26 (AB, total 4H, ^2^J=13.6; Ar—C*H*_2_-Ar); 4.36 and 3.11 (AB, total 4H, ^2^J=13.5; Ar—C*H*_2_-Ar); 3.79 - 3.73 (m, 4H; O—C*H*_2_); 1.93 - 1.85 (m, 4H; propyl), 1.03 (t, 6H, ^3^J=7.5 Hz; propyl). MS (EI) calc. for C_40_H_42_BrNO_6_: m/*z*= 711.22; found m/*z*= 711.1 [*M*]^+^.

### Refinement

H atoms were geometrically positioned and refined using a riding model with fixed individual displacement parameters [U(H) = 1.2 *U*_eq_(C) or U(H) = 1.5 *U*_eq_(C_methyl_)] using a riding model with C—H(aromatic) = 0.95 Å, *C*—*H*(methyl) = 0.98 Å, or *C*—*H*(methylene) = 0.99 Å, respectively. Due to the crystallographic symmetry of the molecule, the Br atom and the nitro group are mutually disordered with equal occupancies. The N atom of the nitro group is so close to the bromine atom that its U value could not be refined and was fixed to 0.05. The following restraints were applied to the nitro group: N—C bond distance 1.470 (1) Å, N—O bond distances 1.220 (1) Å, N···C_α_ distances 2.450 (1) Å. The propenyloxy and propoxy groups are disordered over two sites each with site occupation factors of 0.63 (1) and 0.72 (1), respectively, for the major occupied site. Bond lengths and angles in these groups were restrained to be equal and the displacement ellipsoids of the minor occupied atoms were restrained to an isotropic behaviour.

### Crystal data

**Table:**

C_40_H_42_BrNO_6_	*F*(000) = 1488
*M**_r_* = 712.66	*D*_x_ = 1.313 Mg m^−^^3^
Monoclinic, *C*2/*c*	Mo *K*α radiation, λ = 0.71073 Å
Hall symbol: -C 2yc	Cell parameters from 3120 reflections
*a* = 25.001 (3) Å	θ = 3.5–25.6°
*b* = 8.4963 (14) Å	µ = 1.19 mm^−^^1^
*c* = 19.909 (3) Å	*T* = 173 K
β = 121.530 (8)°	Plate, colourless
*V* = 3604.6 (9) Å^3^	0.26 × 0.12 × 0.11 mm
*Z* = 4	

### Data collection

**Table:**

Stoe IPDS-II two-circle diffractometer	3366 independent reflections
Radiation source: fine-focus sealed tube	1538 reflections with *I* > 2σ(*I*)
graphite	*R*_int_ = 0.081
ω scans	θ_max_ = 25.7°, θ_min_ = 3.5°
Absorption correction: multi-scan (*MULABS*; Spek, 2009; Blessing, 1995)	*h* = −30→25
*T*_min_ = 0.748, *T*_max_ = 0.881	*k* = −10→10
9833 measured reflections	*l* = −24→24

### Refinement

**Table:**

Refinement on *F*^2^	Secondary atom site location: difference Fourier map
Least-squares matrix: full	Hydrogen site location: inferred from neighbouring sites
*R*[*F*^2^ > 2σ(*F*^2^)] = 0.065	H-atom parameters constrained
*wR*(*F*^2^) = 0.130	*w* = 1/[σ^2^(*F*_o_^2^) + (0.0307*P*)^2^] where *P* = (*F*_o_^2^ + 2*F*_c_^2^)/3
*S* = 0.92	(Δ/σ)_max_ < 0.001
3366 reflections	Δρ_max_ = 0.26 e Å^−^^3^
286 parameters	Δρ_min_ = −0.29 e Å^−^^3^
47 restraints	Extinction correction: *SHELXL97* (Sheldrick, 2008), Fc^*^=kFc[1+0.001xFc^2^λ^3^/sin(2θ)]^-1/4^
Primary atom site location: structure-invariant direct methods	Extinction coefficient: 0.0020 (3)

### Special details

**Table:**

Geometry. All e.s.d.'s (except the e.s.d. in the dihedral angle between two l.s. planes) are estimated using the full covariance matrix. The cell e.s.d.'s are taken into account individually in the estimation of e.s.d.'s in distances, angles and torsion angles; correlations between e.s.d.'s in cell parameters are only used when they are defined by crystal symmetry. An approximate (isotropic) treatment of cell e.s.d.'s is used for estimating e.s.d.'s involving l.s. planes.
Refinement. Refinement of *F*^2^ against ALL reflections. The weighted *R*-factor *wR* and goodness of fit *S* are based on *F*^2^, conventional *R*-factors *R* are based on *F*, with *F* set to zero for negative *F*^2^. The threshold expression of *F*^2^ > σ(*F*^2^) is used only for calculating *R*-factors(gt) *etc*. and is not relevant to the choice of reflections for refinement. *R*-factors based on *F*^2^ are statistically about twice as large as those based on *F*, and *R*- factors based on ALL data will be even larger.

### Fractional atomic coordinates and isotropic or equivalent isotropic displacement parameters (Å^2^)

**Table:**

	*x*	*y*	*z*	*U*_iso_*/*U*_eq_	Occ. (<1)
Br1	0.73186 (7)	1.08989 (19)	0.62438 (6)	0.0654 (5)	0.50
N1	0.7175 (3)	1.0605 (11)	0.6027 (4)	0.050*	0.50
O11	0.7045 (4)	1.0828 (14)	0.6532 (4)	0.110 (4)	0.50
O12	0.7628 (3)	1.1127 (11)	0.6024 (5)	0.086 (3)	0.50
O1	0.55519 (13)	0.6827 (4)	0.34459 (15)	0.0402 (8)	
O2	0.58956 (14)	0.6443 (4)	0.19972 (16)	0.0444 (8)	
C1	0.5086 (2)	0.8009 (6)	0.4372 (2)	0.0474 (13)	
H1A	0.5030	0.8392	0.4801	0.057*	
H1B	0.4993	0.6867	0.4303	0.057*	
C2	0.6667 (2)	0.7893 (6)	0.3528 (2)	0.0438 (12)	
H2A	0.6624	0.6752	0.3410	0.053*	
H2B	0.7104	0.8205	0.3711	0.053*	
C11	0.5757 (2)	0.8291 (5)	0.4588 (2)	0.0395 (12)	
C12	0.5960 (2)	0.7747 (5)	0.4091 (2)	0.0356 (11)	
C13	0.6528 (2)	0.8223 (5)	0.4169 (2)	0.0358 (11)	
C14	0.69268 (19)	0.9186 (5)	0.48264 (18)	0.0402 (11)	
H14	0.7317	0.9536	0.4906	0.048*	
C15	0.6751 (2)	0.9614 (4)	0.53478 (18)	0.0416 (12)	
C16	0.61726 (18)	0.9227 (5)	0.5234 (2)	0.0450 (12)	
H16	0.6057	0.9592	0.5592	0.054*	
C17	0.5514 (5)	0.5159 (14)	0.3672 (9)	0.071 (4)	0.625 (13)
H17A	0.5487	0.5163	0.4150	0.086*	0.625 (13)
H17B	0.5128	0.4659	0.3241	0.086*	0.625 (13)
C18	0.6064 (5)	0.4232 (11)	0.3824 (6)	0.066 (4)	0.625 (13)
H18	0.6161	0.4200	0.3422	0.079*	0.625 (13)
C19	0.6430 (7)	0.3450 (13)	0.4472 (7)	0.082 (4)	0.625 (13)
H19A	0.6348	0.3454	0.4888	0.098*	0.625 (13)
H19B	0.6777	0.2877	0.4528	0.098*	0.625 (13)
C17'	0.5755 (9)	0.526 (2)	0.3452 (9)	0.053 (5)	0.375 (13)
H17C	0.6208	0.5247	0.3641	0.064*	0.375 (13)
H17D	0.5522	0.4812	0.2913	0.064*	0.375 (13)
C18'	0.5636 (8)	0.4314 (19)	0.3980 (8)	0.057 (6)	0.375 (13)
H18'	0.5224	0.4326	0.3891	0.068*	0.375 (13)
C19'	0.6059 (12)	0.346 (2)	0.4563 (10)	0.080 (7)	0.375 (13)
H19C	0.6476	0.3419	0.4669	0.096*	0.375 (13)
H19D	0.5949	0.2878	0.4881	0.096*	0.375 (13)
C21	0.62150 (19)	0.8809 (6)	0.2786 (2)	0.0368 (11)	
C22	0.5834 (2)	0.8061 (6)	0.2053 (2)	0.0370 (11)	
C23	0.53622 (19)	0.8876 (6)	0.1391 (2)	0.0404 (12)	
C24	0.5298 (2)	1.0487 (6)	0.1459 (2)	0.0458 (13)	
H24	0.4985	1.1061	0.1018	0.055*	
C25	0.5683 (2)	1.1257 (6)	0.2160 (3)	0.0557 (15)	
H25	0.5639	1.2360	0.2195	0.067*	
C26	0.6134 (2)	1.0433 (6)	0.2817 (2)	0.0450 (13)	
H26	0.6393	1.0982	0.3297	0.054*	
C27	0.6382 (4)	0.6220 (8)	0.1775 (6)	0.038 (2)	0.717 (14)
H27A	0.6793	0.6649	0.2189	0.045*	0.717 (14)
H27B	0.6252	0.6751	0.1270	0.045*	0.717 (14)
C28	0.6421 (3)	0.4457 (8)	0.1700 (5)	0.042 (2)	0.717 (14)
H28A	0.6012	0.4059	0.1268	0.051*	0.717 (14)
H28B	0.6513	0.3941	0.2195	0.051*	0.717 (14)
C29	0.6936 (5)	0.4045 (18)	0.1529 (7)	0.048 (3)	0.717 (14)
H29A	0.6964	0.2898	0.1502	0.072*	0.717 (14)
H29B	0.7339	0.4460	0.1951	0.072*	0.717 (14)
H29C	0.6833	0.4511	0.1025	0.072*	0.717 (14)
C27'	0.6423 (11)	0.562 (4)	0.2081 (11)	0.052 (7)	0.283 (14)
H27C	0.6514	0.4696	0.2427	0.063*	0.283 (14)
H27D	0.6795	0.6318	0.2334	0.063*	0.283 (14)
C28'	0.6306 (10)	0.509 (3)	0.1297 (11)	0.052 (6)	0.283 (14)
H28C	0.6113	0.5955	0.0912	0.062*	0.283 (14)
H28D	0.6007	0.4193	0.1105	0.062*	0.283 (14)
C29'	0.6917 (16)	0.457 (5)	0.135 (2)	0.066 (13)	0.283 (14)
H29D	0.6817	0.3900	0.0901	0.108*	0.283 (14)
H29E	0.7176	0.3979	0.1842	0.108*	0.283 (14)
H29F	0.7147	0.5501	0.1350	0.108*	0.283 (14)

### Atomic displacement parameters (Å^2^)

**Table:**

	*U*^11^	*U*^22^	*U*^33^	*U*^12^	*U*^13^	*U*^23^
Br1	0.0603 (10)	0.0649 (9)	0.0360 (6)	0.0170 (8)	0.0009 (5)	−0.0200 (6)
O11	0.083 (7)	0.167 (10)	0.067 (5)	0.003 (7)	0.029 (5)	−0.061 (6)
O12	0.092 (7)	0.092 (7)	0.059 (5)	−0.022 (6)	0.030 (5)	−0.017 (4)
O1	0.0349 (18)	0.037 (2)	0.0406 (16)	−0.0036 (15)	0.0140 (13)	−0.0072 (13)
O2	0.0324 (19)	0.054 (2)	0.0501 (17)	0.0019 (16)	0.0238 (14)	−0.0162 (14)
C1	0.044 (3)	0.062 (3)	0.038 (2)	−0.004 (3)	0.022 (2)	0.006 (2)
C2	0.028 (3)	0.056 (3)	0.042 (2)	0.008 (2)	0.014 (2)	−0.009 (2)
C11	0.042 (3)	0.045 (3)	0.0243 (19)	0.005 (2)	0.0122 (18)	0.0079 (18)
C12	0.035 (3)	0.034 (3)	0.031 (2)	0.007 (2)	0.0121 (19)	0.0046 (18)
C13	0.035 (3)	0.033 (3)	0.031 (2)	0.009 (2)	0.0109 (18)	0.0032 (18)
C14	0.032 (3)	0.036 (3)	0.036 (2)	0.006 (2)	0.0057 (18)	0.005 (2)
C15	0.048 (3)	0.037 (3)	0.0243 (19)	0.009 (2)	0.0082 (19)	0.0009 (18)
C16	0.045 (3)	0.055 (3)	0.032 (2)	0.017 (3)	0.0178 (19)	0.006 (2)
C17	0.064 (9)	0.043 (7)	0.103 (10)	−0.023 (7)	0.041 (7)	−0.014 (7)
C18	0.083 (8)	0.036 (6)	0.078 (7)	0.001 (6)	0.042 (6)	−0.022 (5)
C19	0.117 (12)	0.035 (6)	0.085 (8)	0.008 (7)	0.047 (8)	0.005 (5)
C17'	0.037 (11)	0.047 (11)	0.068 (10)	0.004 (9)	0.021 (8)	0.005 (7)
C18'	0.063 (12)	0.019 (9)	0.069 (10)	−0.007 (9)	0.021 (9)	−0.004 (7)
C19'	0.103 (18)	0.052 (11)	0.077 (11)	0.015 (12)	0.043 (12)	0.000 (8)
C21	0.029 (2)	0.054 (3)	0.038 (2)	−0.004 (2)	0.0253 (17)	−0.009 (2)
C22	0.038 (3)	0.048 (3)	0.041 (2)	−0.003 (2)	0.031 (2)	−0.009 (2)
C23	0.030 (2)	0.072 (4)	0.031 (2)	−0.003 (3)	0.0232 (18)	−0.005 (2)
C24	0.047 (3)	0.056 (4)	0.039 (2)	0.001 (2)	0.025 (2)	0.004 (2)
C25	0.066 (4)	0.049 (4)	0.051 (3)	−0.008 (3)	0.030 (2)	0.000 (2)
C26	0.043 (3)	0.049 (3)	0.037 (2)	−0.005 (2)	0.017 (2)	−0.006 (2)
C27	0.039 (4)	0.041 (5)	0.049 (5)	0.001 (4)	0.034 (4)	−0.011 (3)
C28	0.035 (4)	0.040 (5)	0.045 (5)	0.000 (4)	0.017 (3)	−0.016 (4)
C29	0.039 (5)	0.054 (9)	0.049 (5)	0.005 (5)	0.022 (4)	−0.016 (6)
C27'	0.053 (13)	0.070 (19)	0.057 (13)	−0.012 (13)	0.045 (12)	0.006 (12)
C28'	0.071 (15)	0.059 (15)	0.043 (11)	0.005 (11)	0.044 (11)	−0.003 (10)
C29'	0.09 (2)	0.05 (2)	0.08 (2)	0.031 (16)	0.059 (16)	−0.006 (16)

### Geometric parameters (Å, °)

**Table:**

Br1—C15	1.934 (3)	C17'—H17C	0.9900
N1—O12	1.2200 (11)	C17'—H17D	0.9900
N1—O11	1.2203 (10)	C18'—C19'	1.307 (16)
N1—C15	1.4704 (10)	C18'—H18'	0.9500
O1—C12	1.390 (5)	C19'—H19C	0.9500
O1—C17'	1.426 (18)	C19'—H19D	0.9500
O1—C17	1.505 (13)	C21—C26	1.401 (6)
O2—C22	1.394 (5)	C21—C22	1.409 (5)
O2—C27'	1.42 (3)	C22—C23	1.408 (6)
O2—C27	1.508 (9)	C23—C24	1.393 (7)
C1—C11	1.518 (6)	C23—C1^i^	1.525 (6)
C1—C23^i^	1.525 (6)	C24—C25	1.377 (6)
C1—H1A	0.9900	C24—H24	0.9500
C1—H1B	0.9900	C25—C26	1.389 (6)
C2—C13	1.514 (6)	C25—H25	0.9500
C2—C21	1.523 (6)	C26—H26	0.9500
C2—H2A	0.9900	C27—C28	1.513 (9)
C2—H2B	0.9900	C27—H27A	0.9900
C11—C16	1.403 (5)	C27—H27B	0.9900
C11—C12	1.404 (6)	C28—C29	1.533 (10)
C12—C13	1.404 (6)	C28—H28A	0.9900
C13—C14	1.418 (5)	C28—H28B	0.9900
C14—C15	1.372 (6)	C29—H29A	0.9800
C14—H14	0.9500	C29—H29B	0.9800
C15—C16	1.380 (5)	C29—H29C	0.9800
C16—H16	0.9500	C27'—C28'	1.500 (16)
C17—C18	1.473 (12)	C27'—H27C	0.9900
C17—H17A	0.9900	C27'—H27D	0.9900
C17—H17B	0.9900	C28'—C29'	1.537 (17)
C18—C19	1.307 (11)	C28'—H28C	0.9900
C18—H18	0.9500	C28'—H28D	0.9900
C19—H19A	0.9500	C29'—H29D	0.9800
C19—H19B	0.9500	C29'—H29E	0.9800
C17'—C18'	1.470 (15)	C29'—H29F	0.9800
			
O12—N1—O11	126.5 (6)	C17'—C18'—H18'	117.7
O12—N1—C15	115.4 (5)	C18'—C19'—H19C	120.0
O11—N1—C15	118.2 (5)	C18'—C19'—H19D	120.0
C12—O1—C17'	115.6 (7)	H19C—C19'—H19D	120.0
C12—O1—C17	112.6 (6)	C26—C21—C22	117.0 (4)
C22—O2—C27'	127.5 (12)	C26—C21—C2	120.8 (4)
C22—O2—C27	106.7 (4)	C22—C21—C2	122.1 (4)
C11—C1—C23^i^	109.5 (4)	O2—C22—C23	118.9 (4)
C11—C1—H1A	109.8	O2—C22—C21	118.9 (4)
C23^i^—C1—H1A	109.8	C23—C22—C21	122.0 (4)
C11—C1—H1B	109.8	C24—C23—C22	118.3 (4)
C23^i^—C1—H1B	109.8	C24—C23—C1^i^	120.7 (4)
H1A—C1—H1B	108.2	C22—C23—C1^i^	121.0 (5)
C13—C2—C21	110.0 (4)	C25—C24—C23	120.7 (4)
C13—C2—H2A	109.7	C25—C24—H24	119.6
C21—C2—H2A	109.7	C23—C24—H24	119.6
C13—C2—H2B	109.7	C24—C25—C26	120.5 (5)
C21—C2—H2B	109.7	C24—C25—H25	119.7
H2A—C2—H2B	108.2	C26—C25—H25	119.7
C16—C11—C12	117.8 (4)	C25—C26—C21	121.3 (4)
C16—C11—C1	121.8 (4)	C25—C26—H26	119.3
C12—C11—C1	120.1 (4)	C21—C26—H26	119.3
O1—C12—C11	118.4 (4)	O2—C27—C28	104.9 (7)
O1—C12—C13	118.4 (4)	O2—C27—H27A	110.8
C11—C12—C13	122.9 (4)	C28—C27—H27A	110.8
C12—C13—C14	116.8 (4)	O2—C27—H27B	110.8
C12—C13—C2	121.2 (4)	C28—C27—H27B	110.8
C14—C13—C2	121.6 (4)	H27A—C27—H27B	108.8
C15—C14—C13	120.1 (4)	C27—C28—C29	110.7 (8)
C15—C14—H14	119.9	C27—C28—H28A	109.5
C13—C14—H14	119.9	C29—C28—H28A	109.5
C14—C15—C16	122.4 (3)	C27—C28—H28B	109.5
C14—C15—N1	119.0 (4)	C29—C28—H28B	109.5
C16—C15—N1	118.5 (4)	H28A—C28—H28B	108.1
C14—C15—Br1	118.4 (3)	C28—C29—H29A	109.5
C16—C15—Br1	119.1 (3)	C28—C29—H29B	109.5
C15—C16—C11	119.6 (4)	H29A—C29—H29B	109.5
C15—C16—H16	120.2	C28—C29—H29C	109.5
C11—C16—H16	120.2	H29A—C29—H29C	109.5
C18—C17—O1	111.6 (10)	H29B—C29—H29C	109.5
C18—C17—H17A	109.3	O2—C27'—C28'	111.2 (19)
O1—C17—H17A	109.3	O2—C27'—H27C	109.4
C18—C17—H17B	109.3	C28'—C27'—H27C	109.4
O1—C17—H17B	109.3	O2—C27'—H27D	109.4
H17A—C17—H17B	108.0	C28'—C27'—H27D	109.4
C19—C18—C17	124.8 (13)	H27C—C27'—H27D	108.0
C19—C18—H18	117.6	C27'—C28'—C29'	111.5 (19)
C17—C18—H18	117.6	C27'—C28'—H28C	109.3
C18—C19—H19A	120.0	C29'—C28'—H28C	109.3
C18—C19—H19B	120.0	C27'—C28'—H28D	109.3
H19A—C19—H19B	120.0	C29'—C28'—H28D	109.3
O1—C17'—C18'	108.2 (15)	H28C—C28'—H28D	108.0
O1—C17'—H17C	110.1	C28'—C29'—H29D	109.5
C18'—C17'—H17C	110.1	C28'—C29'—H29E	109.5
O1—C17'—H17D	110.1	H29D—C29'—H29E	109.5
C18'—C17'—H17D	110.1	C28'—C29'—H29F	109.5
H17C—C17'—H17D	108.4	H29D—C29'—H29F	109.5
C19'—C18'—C17'	124.5 (19)	H29E—C29'—H29F	109.5
C19'—C18'—H18'	117.7		
			
C23^i^—C1—C11—C16	109.7 (5)	C17'—O1—C17—C18	25.5 (12)
C23^i^—C1—C11—C12	−63.7 (6)	O1—C17—C18—C19	125.5 (13)
C17'—O1—C12—C11	−116.6 (9)	C12—O1—C17'—C18'	79.0 (13)
C17—O1—C12—C11	−76.8 (7)	C17—O1—C17'—C18'	−14.8 (11)
C17'—O1—C12—C13	69.9 (9)	O1—C17'—C18'—C19'	−126.8 (19)
C17—O1—C12—C13	109.6 (6)	C13—C2—C21—C26	52.0 (6)
C16—C11—C12—O1	−179.6 (4)	C13—C2—C21—C22	−124.0 (4)
C1—C11—C12—O1	−5.9 (6)	C27'—O2—C22—C23	118.1 (10)
C16—C11—C12—C13	−6.4 (6)	C27—O2—C22—C23	93.9 (5)
C1—C11—C12—C13	167.3 (4)	C27'—O2—C22—C21	−66.3 (11)
O1—C12—C13—C14	178.8 (4)	C27—O2—C22—C21	−90.5 (5)
C11—C12—C13—C14	5.6 (6)	C26—C21—C22—O2	−179.5 (4)
O1—C12—C13—C2	6.3 (6)	C2—C21—C22—O2	−3.4 (6)
C11—C12—C13—C2	−166.9 (4)	C26—C21—C22—C23	−4.1 (6)
C21—C2—C13—C12	65.6 (5)	C2—C21—C22—C23	172.1 (4)
C21—C2—C13—C14	−106.5 (4)	O2—C22—C23—C24	178.8 (4)
C12—C13—C14—C15	−0.2 (6)	C21—C22—C23—C24	3.4 (6)
C2—C13—C14—C15	172.3 (4)	O2—C22—C23—C1^i^	1.5 (6)
C13—C14—C15—C16	−4.3 (6)	C21—C22—C23—C1^i^	−173.9 (4)
C13—C14—C15—N1	179.6 (6)	C22—C23—C24—C25	−0.5 (7)
C13—C14—C15—Br1	178.9 (3)	C1^i^—C23—C24—C25	176.8 (4)
O12—N1—C15—C14	8.1 (12)	C23—C24—C25—C26	−1.4 (7)
O11—N1—C15—C14	−171.8 (9)	C24—C25—C26—C21	0.6 (8)
O12—N1—C15—C16	−168.1 (8)	C22—C21—C26—C25	2.1 (7)
O11—N1—C15—C16	11.9 (13)	C2—C21—C26—C25	−174.1 (4)
C14—C15—C16—C11	3.5 (6)	C22—O2—C27—C28	−178.5 (6)
N1—C15—C16—C11	179.6 (6)	C27'—O2—C27—C28	42.4 (18)
Br1—C15—C16—C11	−179.8 (3)	O2—C27—C28—C29	−176.1 (7)
C12—C11—C16—C15	1.8 (6)	C22—O2—C27'—C28'	−105 (2)
C1—C11—C16—C15	−171.8 (4)	C27—O2—C27'—C28'	−52.8 (17)
C12—O1—C17—C18	−77.5 (10)	O2—C27'—C28'—C29'	166 (2)

Symmetry codes: (i) −*x*+1, *y*, −*z*+1/2.
